# Hyperosmolar sodium-lactate in the ICU: vascular filling and cellular feeding

**DOI:** 10.1186/s13054-014-0599-5

**Published:** 2014-11-07

**Authors:** Eric Fontaine, Jean-Christophe Orban, Carole Ichai

**Affiliations:** University Grenoble Alpes, LBFA, F-38000 Grenoble, France; INSERM, U1055, F-38000 Grenoble, France; Medicosurgical Intensive Care Unit, Saint Roch University Hospital, 5 rue Pierre Dévoluy, 06000 Nice, France; Nice Sophia-Antipolis University, F-06000 Nice, France; IRCAN Unit (Inserm U1081, CNRS UMR7284), Nice Sophia-Antipolis University, 06000 Nice, France

## Abstract

Hyperosmolar lactate-based solutions have been used for fluid resuscitation in ICU patients. The positive effects observed with these fluids have been attributed to both lactate metabolism and the hypertonic nature of the solutions. In a recent issue of *Critical Care*, Duburcq and colleagues studied three types of fluid infused at the same volume in a porcine model of endotoxic shock. The control group was resuscitated with 0.9% NaCl, and the two other groups received either hypertonic sodium-lactate or hypertonic sodium-bicarbonate. The two hypertonic fluids proved to be more effective than 0.9% NaCl for resuscitation in this model. However, some parameters were more effectively corrected by hypertonic sodium-lactate than by hypertonic sodium-bicarbonate, suggesting that lactate metabolism was beneficial in these cases.

Glucose is an energy substrate because its catabolism into CO_2_ and H_2_O allows the synthesis of ATP. During this process, glucose is first converted into pyruvate (glycolysis) before pyruvate enters mitochondria to be further catabolized (via the citric acid cycle). When pyruvate does not enter mitochondria, it leaves the cell in which it was produced to enter another one where it is converted into glucose (gluconeogenesis) or catabolized to allow ATP production. Pyruvate can also be converted into lactate in all cells. Importantly, the reaction is reversible; therefore, both glucose and lactate can be metabolized into pyruvate. Moreover, lactate leaves or enters the cell via the same transporter as pyruvate. Depending on the distance between the cells that release them and those that take them up, pyruvate and lactate travel, or not, in blood.

If from a biochemical point of view pyruvate and lactate are carbohydrates perfectly usable for ATP production, their metabolism has some particularities. Compared with glucose, pyruvate and lactate are easily metabolized by all tissues even in the case of insulin resistance because insulin does not regulate the pyruvate-lactate transporter.

In ICU patients, both blood glucose [1] and lactate [2] concentrations positively correlate with the severity of illness. Does this mean that glucose and lactate worsen disease in the critically ill? The answer remains uncertain for glucose, considering the efforts made to control blood glucose in ICU patients. However, this does not preclude using glucose in the ICU. Is lactate toxic or harmful? Growing evidence convincingly suggests that this is not the case. Lactate metabolism has been studied in both volunteers and patients by measuring lactate clearance after sodium-lactate loads that increased blood lactate concentrations above 10 mmol/L [3,4]. No side effects were observed, except an expected alkalinization and decrease in blood potassium concentration. Indeed, sodium-lactate has been used instead of sodium-bicarbonate to treat ventricular tachycardia [5]. Although counterintuitive, pioneer studies have shown the harmlessness and usefulness of sodium-lactate in ICU patients. Hyperosmolar sodium-lactate has been shown (i) to improve cardiac performance in patients undergoing elective cardiac surgery [6] and during acute heart failure [7]; (ii) to induce a negative cumulative fluid balance after coronary artery bypass grafting [8] and to decrease fluid accumulation during burn shock resuscitation [9]; (iii) to be effective in treating [10] and preventing [11] intracranial hypertension following severe traumatic brain injury; and (iv) to restore hemodynamic status in dengue shock syndrome with minimal fluid accumulation [12].

The mechanism by which hyperosmolar sodium-lactate improved these parameters was attributed to both lactate metabolism and hyperosmolarity, but no discrimination between these two mechanisms was made. In a recent study published in *Critical Care*, Duburcq and colleagues elegantly discriminated between these two mechanisms using a porcine model of endotoxic shock [13]. Once shock had been established, the animals received the same volume of three different crystalloid-based solutions: 0.9% NaCl (control group), hyperosmolar sodium-bicarbonate or hyperosmolar sodium-lactate. Importantly, the hyperosmolar groups received the same amount of sodium and osmoles. Duburcq and colleagues observed that both hyperosmolar sodium-bicarbonate and hyperosmolar sodium-lactate prevented anuria and improved mean pulmonary arterial pressure, mixed venous oxygen saturation, oxygen extraction and oxygen delivery/global oxygen consumption ratio better than the control solution. However, hyperosmolar sodium-lactate improved mean arterial pressure, microvascular reactivity, arterial oxygen partial pressure/inspired oxygen fraction ratio, blood glucose concentration and fluid balance better than hyperosmolar sodium-bicarbonate and the control solution. This suggests that some of the positive effects of hyperosmolar sodium-lactate are due to the hyperosmolar nature of the solution, while others are directly related to lactate metabolism.

Both sodium-bicarbonate and sodium-lactate are metabolizable anions. This means that the negative charge (bicarbonate or lactate) is converted into uncharged compounds, resulting in sodium load without chloride. In this respect, sodium-bicarbonate and sodium-lactate are responsible for similar effects on pH and electrolytes. Contrary to bicarbonate, however, lactate is an energy substrate (3.61 kcal/g). Therefore, future experiments could use hyperosmolar sodium-bicarbonate supplemented with another energy substrate (glucose, for example) in order to further specify whether the advantage of lactate is related to its particular metabolism or simply to its energy load.

## References

[1] Krinsley JS (2003). Association between hyperglycemia and increased hospital mortality in a heterogeneous population of critically ill patients. Mayo Clin Proc.

[2] Vincent JL (1995). Lactate levels in critically ill patients. Acta Anaesthesiol Scand Suppl.

[3] Chiolero R, Tappy L, Gillet M, Revelly JP, Roth H, Cayeux C, Schneiter P, Leverve X (1999). Effect of major hepatectomy on glucose and lactate metabolism. Ann Surg.

[4] Chiolero RL, Revelly JP, Leverve X, Gersbach P, Cayeux MC, Berger MM, Tappy L (2000). Effects of cardiogenic shock on lactate and glucose metabolism after heart surgery. Crit Care Med.

[5] Scanu P, Grollier G, Guilleman D, Iselin M, Bustany P, Hurpe JM, Potier JC (1991). Malignant ventricular tachycardia during propafenone treatment in a child with junctional automatic tachycardia: effectiveness of intravenous molar sodium lactate. Pacing Clin Electrophysiol.

[6] Mustafa I, Leverve XM (2002). Metabolic and hemodynamic effects of hypertonic solutions: sodium-lactate versus sodium chloride infusion in postoperative patients. Shock.

[7] Nalos M, Leverve X, Huang S, Weisbrodt L, Parkin R, Seppelt I, Ting I, McLean A (2014). Half-molar sodium lactate infusion improves cardiac performance in acute heart failure: a pilot randomised controlled clinical trial. Crit Care.

[8] Leverve XM, Boon C, Hakim T, Anwar M, Siregar E, Mustafa I (2008). Half-molar sodium-lactate solution has a beneficial effect in patients after coronary artery bypass grafting. Intensive Care Med.

[9] Belba MK, Petrela EY, Belba GP (2009). Comparison of hypertonic vs isotonic fluids during resuscitation of severely burned patients. Am J Emerg Med.

[10] Ichai C, Armando G, Orban JC, Berthier F, Rami L, Samat-Long C, Grimaud D, Leverve X (2009). Sodium lactate versus mannitol in the treatment of intracranial hypertensive episodes in severe traumatic brain-injured patients. Intensive Care Med.

[11] Ichai C, Payen JF, Orban JC, Quintard H, Roth H, Legrand R, Francony G, Leverve XM (2013). Half-molar sodium lactate infusion to prevent intracranial hypertensive episodes in severe traumatic brain injured patients: a randomized controlled trial. Intensive Care Med.

[12] Somasetia DH, Setiati TE, Sjahrodji AM, Idjradinata PS, Setiabudi D, Roth H, Ichai C, Fontaine E, Leverve XM (2014). Early resuscitation of Dengue Shock Syndrome in children with hyperosmolar sodium-lactate: a randomized single blind clinical trial of efficacy and safety. Crit Care.

[13] Duburcq T, Favory R, Mathieux D, Hubert T, Mangalaboyi J, Gmyr V, Quintane L, Maboudou P, Pattou F, Jourdain M (2014). Hypertonic sodium lactate improves fluid balance and hemodynamics in porcine endotoxic shock. Crit Care.

